# Liquid Chromatographic Determination of Alogliptin in Bulk and in its Pharmaceutical Preparation

**Published:** 2012-09

**Authors:** Ramzia I. El-Bagary, Ehab F. Elkady, Bassam M. Ayoub

**Affiliations:** *Department of Pharmaceutical Chemistry, Faculty of Pharmacy, Cairo University, Kasr El-Aini St., Cairo 11562, Egypt*

**Keywords:** alogliptin, reversed-phase liquid chromatography, isocratic elution, pharmaceutical preparation

## Abstract

In this work, a reversed-phase liquid chromatographic (RP-LC) method has been developed for the determination of alogliptin (ALG) based on isocratic elution using a mobile phase consisting of potassium dihydrogen phosphate buffer pH (4.6)-acetonitrile (20:80, *v/v*) at a flow rate of 1 mL min^−1^ with UV detection at 215 nm. Chromatographic separation was achieved on a Symmetry^®^ cyanide column (150 mm × 4.6 mm, 5 μm). Linearity, accuracy and precision were found to be acceptable over the concentration range of 5-160 μg mL^−1^ for ALG in bulk. The optimized method was validated and proved to be specific, robust and accurate for the quality control of ALG in pharmaceutical preparations.

## INTRODUCTION

**Figure 1:**
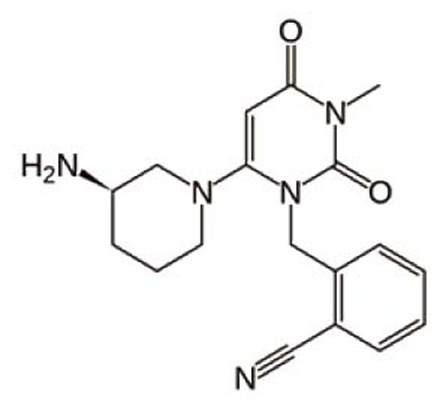
Chemical structure of alogliptin.

Alogliptin (ALG), 2-({6-[(3*R*)-3-aminopiperidin-1-yl]-3-methyl-2,4-dioxo-3,4-dihydropyrimidin-1(2*H*)-yl}methyl)benzonitrile (Fig. 1) is a novel hypoglycemic drug that belongs to dipeptidyl-peptidase-4 inhibitor class which stimulates glucose-dependent insulin release (1, 2). DPP-4 inhibitors represent a new therapeutic approach to the treatment of type 2 diabetes that functions to stimulate glucose-dependent insulin release and reduce glucagons levels. This is done through inhibition of the inactivation of incretins, particularly glucagon-like peptide-1 (GLP-1) and gastric inhibitory polypeptide (GIP), thereby improving glycemic control (3). Recently, DPP-4 inhibitors have been recommended in the treatment of diabetes mellitus to improve glycemic control (4) and it is effective in controlling the metabolic syndrome and resulted in significant weight loss, a reversal of insulin resistance, islet and adipocyte hypertrophy, and alleviated hepatic steatosis (5). To the best of authors’ knowledge, there are not any methods that have been described for the determination of ALG in bulk or pharmaceutical preparation. Thus, the aim of the present work was to develop a LC method for the determination of ALG in bulk and pharmaceutical preparation applying UV detection.

## EXPERIMENTAL

### Instrumentation

The HPLC system consisted of a Schimadzu LC-20 AT Liquid Chromatograph (Japan) using a Symmetry^®^ cyanide column (150 mm × 4.6 mm, 5 μm). The system was equipped with a UV-visible detector (SPD-20A, Japan) and an autosampler (SIL-20A, Schimadzu, Japan). An Elma S100 ultrasonic processor model KBK 4200 (Germany) was used.

### Reagents and reference samples

Pharmaceutical grade ALG, certified to contain 99.70%, Nesina^®^ tablets nominally containing 25 mg of ALG per tablet were supplied from Takeda pharmaceutical company (Japan). HPLC grade acetonitrile and methanol were purchased from Fisher Scientific (Loughborough, Leicestershire, UK). Potassium dihydrogen phosphate and orthophosphric acid (85%) were purchased from VWR Chemicals (Pool, England). Bi-distilled water was produced in-house (Aquatron Water Still, A4000D, UK). Membrane filters 0.45 μm from Teknokroma (Barcelona, Spain) were used. All other chemicals and reagents used were of analytical grade unless indicated otherwise. Standard stock solution of ALG (1 mg mL^-1^) was prepared by dissolving 100 mg of ALG in methanol in a 100 mL volumetric flask and completing to volume with methanol and then the required concentrations were prepared by serial dilutions.

### Chromatographic conditions

Chromatographic separation was achieved on a Symmetry^®^ cyanide column (150 mm × 4.6 mm, 5 μm) applying an isocratic elution based on potassium dihydrogen phosphate buffer pH (4.6)-acetonitrile (20:80, *v/v*) as a mobile phase. The UV detector was operated at 215 nm. The buffer solution was filtered through 0.45 μm membrane filter and degassed for 30 min in an ultrasonic bath prior to its use. The mobile phase was pumped through the column at a flow rate of 1 mL min^-1^. Analyses were performed at ambient temperature and the injection volume was 25 μL.

### Sample preparation

Twenty tablets of Nesina^®^ were weighed, powdered and mixed in a mortar. An accurately weighed amount of the finely powdered Nesina^®^ tablets equivalent to 100 mg of ALG were made up to 100 mL with methanol and sonicated to dissolve. The solutions were filtered followed by serial dilutions to the required concentrations for each experiment.

### Procedure

**Linearity and repeatability.** Accurately measured aliquots of stock solutions equivalent to 50-1600 μg ALG were transferred into a series of 10 mL volumetric flasks and then completed to volume with methanol. A volume of 25 μL of each solution was injected into the chromatograph. The conditions including the mobile phase at a flow rate 1 mL min^-1^, detection at 215 nm and run time program for 10 min were adjusted. A calibration curve was obtained by plotting area under the peak (AUP) against concentration (C). The repeatability of the method was assessed by analyzing 50 μg mL^-1^ of ALG (*n*=6). The precision (%R.S.D) values of peak areas and retention times were calculated, Table 1.

**Table 1:** System suitability tests for LC-UV method for the determination of alogliptin in bulk

Item	ALG

N	951
T	1.01
RSD% of 6 injections	
Peak area	0.55
Retention time	0.31

A**ssay of ALG in bulk and Nesina^®^ tablets.** The procedure mentioned under **Linearity and repeatability** was repeated using concentrations equivalent to 15-135 μg mL^-1^ ALG in bulk. For the determination of ALG in Nesina^®^ tablets, the sample solution prepared under **Sample preparation** was serially diluted and then injected in triplicates. The concentrations of ALG were calculated using calibration equation.

## RESULTS AND DISCUSSION

HPLC greatly reduces the analysis time and allows for the determination of many individual components in a mixture using one single procedure (6). We applied the technique of detection widely applied in routine analysis; namely UV detection.

### Method development

**Figure 2:**
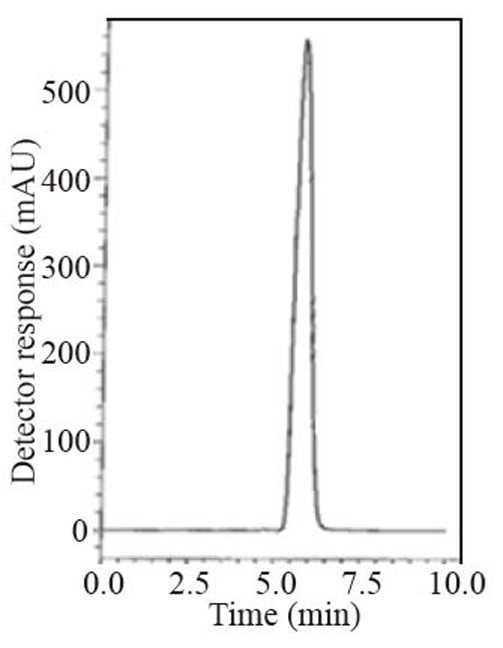
A typical LC chromatogram with ultraviolet detection of 25 μL injector of alogliptin in bulk sample solution (50 μg mL^-1^).

**Figure 3:**
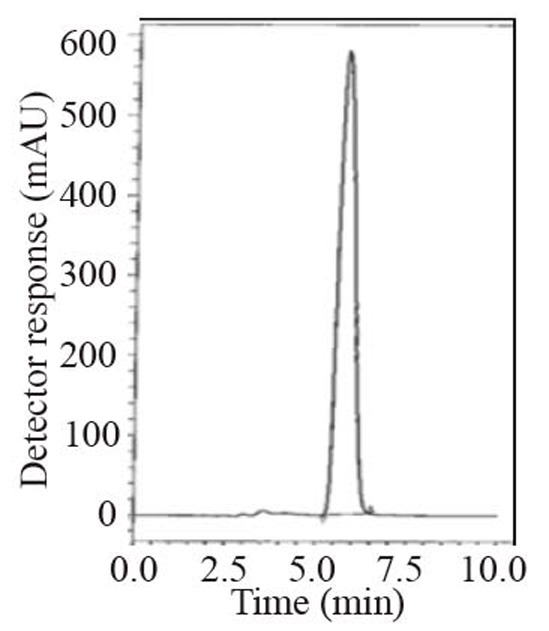
A typical LC chromatogram with ultraviolet detection of 25 μL injector of Nesina^®^ sample solution (50 μg mL^-1^).

During the optimization cycle, several chromatographic conditions were attempted using Symmetry^®^ cyanide column (150 mm × 4.6 mm, 5 μm). Various mobile phase compositions containing different ratios of organic and aqueous phases were tried in an isocratic mode. Acetonitrile was found optimum for the elution. Besides, different buffers at different pH values were attempted along with acetonitrile. Therefore, a mobile phase consisting of potassium dihydrogen phosphate buffer pH (4.6) - acetonitrile (20:80, *v/v*) and pumped at a flow rate of 1.0 mL min^-1^, in an isocratic mode, gave good result. Detection was carried out at 215 nm. The retention time was 5.8 min for ALG as in Fig. 2 and Fig. 3.

### System suitability tests

According to USP 2007 (7), system suitability tests are an integral part of liquid chromatographic methods in the course of optimizing the conditions of the proposed method. System suitability tests are used to verify that the resolution and reproducibility were adequate for the analysis performed. The parameters of these tests are column efficiency (number of theoretical plates), tailing of chromatographic peak and repeatability as %R.S.D of peak area for six injections and reproducibility of retention as %R.S.D of retention time. The results of these tests are listed in Table 1.

### Method validation

**Linearity.** Linearity was studied for ALG. A linear relationship between area under the peak (AUP) and concentration (C) was obtained. The regression equation was also computed, Table 2. The linearity of the calibration curve was validated by the high value of correlation coefficient. The analytical data of the calibration curve including standard deviation for the slope and intercept (S_b_, S_a_) are summarized in Table 2.

**Table 2:** Results obtained for LC-UV method for the determination of alogliptin in bulk

Item	Alogliptin

Retention time	5.8
Wavelength of detection	215 nm
Range of linearity	5-160 μg mL^-1^
Regression equation	Area × 10^-5^ = 1.1068 C_μg mL^-1^_ - 0.5600
Regression coefficient (r^2^)	0.9999
LOD (μg mL^-1^)	1.07
LOQ (μg mL^-1^)	3.57
S_b_	1.6 × 10^-3^
S_a_	0.15
Confidence limit of the slope	1.1068 ± 0.17
Confidence limit of the intercept	-0.5600 ± 0.9 × 10^-3^
Standard error of the estimation	0.21
Precision	
Intraday %R.S.D	0.24-0.66
Interday %R.S.D	0.22-1.34
Drug in bulk	100.34 ± 0.71
Drug in dosage form	100.06 ± 1.48
Drug added	100.14 ± 0.73

**Accuracy.** Accuracy of the results was calculated by % recovery of 5 different concentrations of ALG and also by standard addition technique applied for Nesina^®^ tablets, all carried out in triplicates. The results obtained including the mean of the recovery and standard deviation are displayed in Table 2.

**Precision.** The repeatability of the method was assessed by six determinations for each of the three concentrations of ALG (40-50-60 μg.ml^-1^) representing 80-100-120%, respectively. The repeatability of sample application and measurement of peak area of active compound were expressed in terms of percentage relative standard deviation (%R.S.D.) and found to be less than 1% in the three concentrations. Results for the determination of precision are displayed in Tables 1, 2.

**Specificity.** Specificity is the ability of the analytical method to measure the analyte response in the presence of interferences including degradation products and related substances. The chromatograms of the samples were checked for the appearance of any extra peaks. No chromatographic interference from any of the excipients was found at the retention times of the examined compounds (Fig. 2). In addition, the chromatogram of each compound in the sample solution was found identical to the chromatogram received by the standard solution at the wavelength applied. These results demonstrate the absence of interference from other materials in the pharmaceutical formulations and therefore confirm the specificity of the proposed methods.

**Limit of detection and limit of quantification.** Limit of detection (LOD) which represents the concentration of analyte at S/N ratio of 3 and limit of quantification (LOQ) at which S/N is 10 were determined experimentally for the proposed methods and results are given in Table 2.

**Statistical analysis.** A statistical analysis of the results obtained by the proposed method and the reference method was carried out by “SPSS statistical package version 11”. The significant difference between groups was tested by one way ANOVA (F-test) at *p*=0.05 as shown in Table 3. The test ascertained that there was no significant difference among the methods.

**Table 3:** Statistical comparison between the proposed method and the reference method for the determination of alogliptin

Statistical Term	Reference Method^b^	HPLC method

Mean	100.59	100.34
S.D.±	1.46	0.71
S.E. ±	0.65	0.32
%RSD	1.45	0.71
n	5	5
V	2.13	0.50
t (a2.306)		0.34

a Figures in parentheses are the theoretical t value at (*p*=0.05). No significant difference between groups by using one way ANOVA with F equals 0.12 and p equals 0.74;

b Reference method: aliquots of standard solutions in methanol containing 1-9 μg/ml ALG were measured at 215 nm using methanol as a blank (2).

## CONCLUSION

The proposed LC method proved to be simple, accurate and reproducible for the determination of ALG in a reasonable run time. The method was validated showing satisfactory data for all the method validation parameters tested. The developed method can be conveniently used by quality control laboratories.
